# Quantifying Bias in Randomized Controlled Trials in Child Health: A Meta-Epidemiological Study

**DOI:** 10.1371/journal.pone.0088008

**Published:** 2014-02-04

**Authors:** Lisa Hartling, Michele P. Hamm, Ricardo M. Fernandes, Donna M. Dryden, Ben Vandermeer

**Affiliations:** 1 Alberta Research Centre for Health Evidence, Department of Pediatrics, University of Alberta, Edmonton, Alberta, Canada; 2 Clinical Pharmacology Unit, Faculty of Medicine, Instituto de Medicina Molecular, University of Lisbon, Lisbon, Portugal; 3 Department of Pediatrics, Santa Maria Hospital, Lisbon, Portugal; Canadian Agency for Drugs and Technologies in Health, Canada

## Abstract

**Objective:**

To quantify bias related to specific methodological characteristics in child-relevant randomized controlled trials (RCTs).

**Design:**

Meta-epidemiological study.

**Data Sources:**

We identified systematic reviews containing a meta-analysis with 10–40 RCTs that were relevant to child health in the Cochrane Database of Systematic Reviews.

**Data Extraction:**

Two reviewers independently assessed RCTs using items in the Cochrane Risk of Bias tool and other study factors. We used meta-epidemiological methods to assess for differences in effect estimates between studies classified as high/unclear vs. low risk of bias.

**Results:**

We included 287 RCTs from 17 meta-analyses. The proportion of studies at high/unclear risk of bias was: 79% sequence generation, 83% allocation concealment, 67% blinding of participants, 47% blinding of outcome assessment, 49% incomplete outcome data, 32% selective outcome reporting, 44% other sources of bias, 97% overall risk of bias, 56% funding, 35% baseline imbalance, 13% blocked randomization in unblinded trials, and 1% early stopping for benefit. We found no significant differences in effect estimates for studies that were high/unclear vs. low risk of bias for any of the risk of bias domains, overall risk of bias, or other study factors.

**Conclusions:**

We found no differences in effect estimates between studies based on risk of bias. A potential explanation is the number of trials included, in particular the small number of studies with low risk of bias. Until further evidence is available, reviewers should not exclude RCTs from systematic reviews and meta-analyses based solely on risk of bias particularly in the area of child health.

## Introduction

While randomized controlled trials (RCTs) are considered to be the gold standard for evidence on therapeutic interventions, [1] they are nonetheless susceptible to bias. [2] Bias, or the systematic over- or under-estimation of a treatment’s effect, has important implications for decision-making. The implications stem from false positive and false negative results. In practice this may result in the implementation of interventions that are not efficacious and potentially harmful, or withholding of interventions that truly are efficacious. The types of bias that may occur in RCTs can generally be classified as selection, performance, detection, attrition, and reporting bias. [3] The extent to which these biases operate in a given trial may yield inaccuracies of varying magnitude and direction in the estimates of a treatment’s effect.

There is a growing body of empirical evidence based on meta-epidemiological methods to quantify different biases in RCTs; however, there are some inconsistencies across studies and clinical areas. Biases may vary across different clinical areas and investigation within different areas is warranted. [4], [5] Balk et al. found variation in the direction of effects across studies which “calls into question whether any of these associations could provide a general rule for evaluating RCTs across clinical areas.” [4] Furthermore, the evidence to date has stemmed primarily from examination of trials involving adult participants; no meta-epidemiological studies have focused specifically on pediatric trials. Research in children presents specific methodological and practical challenges, such as generating adequate sample sizes, and use of surrogate outcomes or outcome tools that have not been validated for the pediatric population. [6], [7] A meta-epidemiological study to quantify bias in a sample of pediatric trials would better inform the design, conduct, reporting, and interpretation of research in child health.

The goal of this project was to quantify the extent of bias related to specific methodological characteristics in child-relevant RCTs. This will allow for more informed appraisal and application of research findings to patient care, thus providing children with the most appropriate interventions to optimize health outcomes. Our specific objectives were to measure the association between pre-specified methodological characteristics and treatment effect estimates, and explore variations based on different analytic approaches and types of outcomes.

## Methods

We conducted a meta-epidemiological study based on a sample of RCTs contributing to the meta-analyses identified within child-relevant systematic reviews (SRs).

### Study Sample

The sampling frame was the Cochrane Database of Systematic Reviews (CDSR). As part of ongoing work through the Cochrane Child Health Field, child-relevant SRs in the CDSR are identified. A total of 793 child-relevant SRs were considered for eligibility in the present study. These reviews have been previously described. [8] The CDSR was chosen for the sampling frame because: 1) Cochrane reviews provide tabulated data from the component trials as well as detailed descriptions of key characteristics (e.g., study population); 2) Cochrane reviews provide a detailed list of references for all relevant trials; 3) Cochrane reviews have been reported to be of higher methodological quality[9]–[13] which may translate into more comprehensive searches, hence more variability with respect to methodological characteristics; 4) the CDSR offers a more homogeneous sample with respect to domains (i.e., therapeutic effectiveness) and study design (i.e., focus on RCTs).

SRs were included if they: 1) contained a minimum of five RCTs[5]; [14] involving only pediatric patients (ages 0 to 17 years), and a maximum of 40 RCTs, [15] that contribute to at least one meta-analysis; and 2) addressed a question of therapeutic effectiveness. Further, the RCTs in the SRs must have been: superiority studies with parallel designs involving at least two comparison groups; and, reported in “full-length”. [16], [17] Trials that appeared in more than one meta-analysis were retained in the meta-analysis that was randomly selected. From the full sample of child-relevant SRs, 424 (53%) had no meta-analysis and 302 (38%) had meta-analyses with fewer than five studies. From the remaining 68 systematic reviews, we selected those with the largest numbers of studies included in order to optimize the power to detect differences. In particular, we wanted to optimize the chances of having sufficient numbers of studies with low risk of bias as this was the reference category for the analyses. We know from previous work that the vast majority of pediatric trials are at high or unclear risk of bias. [18], [19] We included meta-analyses until we met our intended sample size.

### Data Extraction

Data from the meta-analysis that corresponded to the primary outcome in each SR were extracted. For binary outcomes, the numbers in each group with or without the event and the total number in each group were extracted. For continuous outcomes, the mean and standard deviation for each group was extracted. The outcomes were categorized as objective or subjective based on previously reported criteria. [20].

The following methodological characteristics were assessed for each trial: sequence generation; allocation concealment; blinding of study personnel/participants; blinding of outcome assessment; incomplete outcome reporting; selective outcome reporting; baseline imbalance; trials stopped early for benefit; blocked randomization in unblinded trials; inappropriate influence of trial sponsors; and, sample size. Each methodological characteristic was assessed as high, unclear, or low risk of bias based on guidelines for applying the Cochrane Risk of Bias tool. [3] For selective outcome reporting, we compared the presented results with the outcomes mentioned in the methods section of the same article. [21], [22] Sample size was categorized as large (low risk; minimum 200 patients across two groups[1], [23], [24]) and small (high risk; less than 200 patients).

In addition to the methodological characteristics of interest, the following study characteristics were extracted for each trial: year of publication; publication status; single versus multi-centre; type of intervention; [17] type of control; [4] completeness of outcome reporting; [21] and, source of funding.

A data extraction form and instruction manual were developed to capture study characteristics, methodological characteristics (i.e., risk of bias), and outcome data. The data extraction form was pilot tested by all members of the study team using five trials and revisions were made. One individual independently extracted data from each trial and a second individual checked for completeness and accuracy. Discrepancies were resolved through discussion.

### Data Analysis

The RCTs were described in terms of the study characteristics and methodological characteristics listed above using frequencies and percentages.

For continuous data, a standardized mean difference (SMD) was computed for each study and pooled within each meta-analysis. Outcomes were coded such that higher results were undesirable, thus an SMD of less than zero suggests treatment is beneficial. For dichotomous data, endpoints were re-coded, as necessary, so that the outcome occurrence was undesired (e.g., death rather than survival); hence, an odds ratio of less than one suggests that the treatment is beneficial. For each trial, we calculated a log odds ratio and standard error of the odds ratio for the effect of treatment on the binary outcome of interest. [17] Within each meta-analysis results were pooled using a random effects method.

The pooled results of all the meta-analyses were then combined in a “meta-meta” analysis, using an inverse variance random effects method and subgrouped by the different risk of bias components. For dichotomous data, odds ratios were converted to SMDs using the methods proposed by Hasselblad and Hedges [25] in order to allow us to combine both dichotomous and continuous meta-analyses. A “difference of differences” was then computed between the two subgrouped categories (e.g. low versus unclear or high risk of bias) [26] in order to ascertain differences in results based on the various risk of bias components. A priori, we planned a sensitivity analysis comparing studies at low or unclear vs. high risk of bias. We also conducted meta-regression analyses for each risk of bias component with the individual risk of bias categories (high, unclear, low) as independent variables.

Analyses were performed using Review Manager version 5.0 (Nordic Cochrane Centre, The Cochrane Collaboration, Copenhagen) and Stata version 7.0 (Stata Corporation, College Station, Texas). The raw data used for these analyses are available from authors on request.

There are few precedents in the literature for calculating sample sizes in meta-epidemiological studies. [27] Two previous methodological studies based their sample size on anticipated workload [15] and time constraints. [28] Sample size for another study was based on the sample size used in a previous similar study. [16] We used a pragmatic approach to sample size. The previous meta-epidemiological studies, exclusive of meta-meta-epidemiological research, had sample sizes ranging from 127 to 523 trials (median 220, inter-quartile range 158, 282) from 11 to 48 SRs (median 20, inter-quartile range 14, 32). [17] Therefore, we planned for a sample size of 300 trials.

## Results

Meta-analyses from 17 SRs, comprising 287 studies, were included in the study sample (Table 1). The SRs covered a range of topics which included both drug (n = 7) and non-drug (n = 10) interventions. Comparisons also varied and included placebo or no intervention (n = 9), another active intervention (n = 2), or mixed comparators (n = 6). The outcomes were considered objective in 11 and subjective in 6 of the included meta-analyses. The number of studies included in the meta-analyses ranged from 10 to 32 with a median of 13. 155 RCTs were conducted at a single center, 112 were conducted at multiple centers, and in 20 trials the number of centers was not reported or was unclear. The majority of trials were published (97%) and year of publication ranged from 1965 to 2010 (median 1995). Sources of funding for the trials included: government (n = 73), pharmaceutical industry (n = 33), multiple sources (n = 29), other (n = 7), and unclear or not reported (n = 131).

**Table 1:** Description of meta-analyses included in sample.

Topic area	Interventiontype	Comparisontype	Outcome	Outcome classification	Number of studies	Year of publication (range)	Single-centre/multi-centre*
Glucocorticoids for croup	Drug	Placebo	Clinical score	Subjective	12	1982–1999	10/2
Antibiotics for the prevention of acute and chronicsuppurative otitis media in children	Drug	Placebo	Prevention of AOM or CSOM	Objective	14	1972–2008	6/8
Chemoprophylaxis and intermittent treatment for preventing malaria in children	Drug	Placebo	Clinical malaria	Objective	10	1993–2007	4/6
Immunostimulants for preventing respiratory tract infection in children	Drug	Placebo	Acute respiratory tract infections	Objective	26	1976–2004	10/7
Interventions for educating children who are at risk of asthma-related emergency department attendance	Non-drug	Mixed	ED visits	Objective	17	1986–2006	11/5
Oral versus intravenous rehydration for treating dehydrationdue to gastroenteritis in children	Non-drug	Active intervention	Failure to rehydrate	Objective	12	1982–1995	10/2
Oral zinc for treating diarrhoea in children	Drug	Mixed (mostly placebo)	Diarrhoea duration	Objective	13	1998–2010	8/5
Polymer-based oral rehydration solution for treating acute water diarrhoea	Non-drug	Active intervention	Diarrhoea duration	Objective	11	1982–2001	11/0
Probiotics for treating acute infectious diarrhoea	Non-drug	Mixed (mostly placebo)	Diarrhoea duration	Objective	33	1994–2009	26/6
Rotavirus vaccine for preventing diarrhoea	Non-drug	Placebo	Episodes of diarrhoea	Objective	19	1987–1997	11/8
School-based secondary prevention programmes for preventing violence	Non-drug	Mixed (mostly no intervention)	Aggression	Subjective	32	1977–2001	14/17
Alarm interventions for nocturnal enuresis in children	Non-drug	No intervention	Nights without bedwetting	Objective	13	1973–2002	12/1
Desmopressin for nocturnal enuresis in children	Drug	Placebo	Nights with bedwetting	Objective	10	1978–2001	1/6
Tricyclic drugs for depression in children and adolescents	Drug	Placebo	Depression	Subjective	12	1981–2001	9/3
Cognitive behavioural therapy for anxiety disorders inchildren and adolescents	Non-drug	No intervention	Anxiety	Subjective	12	1994–2003	8/4
Fluoride gels for preventing dental caries inchildren and adolescents	Non-drug	Mixed	D(M)FS increment	Subjective	12	1970–1999	1/9
Fluoride mouth rinses for preventing dental caries in children and adolescents	Non-drug	Mixed (mostly placebo)	D(M)FS increment	Subjective	29	1967–1998	3/23

* where totals do not equal number of studies, the balance of studies did not report this variable.

The methodological quality or risk of bias of the included trials is described in Table 2. The majority of trials were unclear for sequence generation (76%) and allocation concealment (79%). Two-thirds of studies were high or unclear risk for blinding of participants and personnel; approximately half were high or unclear risk for blinding of outcome assessment. The majority of studies were considered low risk for incomplete outcome data, selective outcome reporting, and other sources of bias. Less than half of trials were low risk for source of funding. The majority of trials were low risk for bias associated with baseline imbalances. Among 58 trials that used blocked randomization, there was an equal distribution among high, unclear, and low risk of bias. Five trials reported stopping early for benefit and there was an equal distribution across risk of bias categories.

**Table 2:** Risk of bias assessments by domain (N = 287).

Domain	Risk of bias assessments – n (%)		
	High	Unclear	Low
Sequence generation	11 (3.8)	217 (75.6)	59 (20.6)
Allocation concealment	12 (4.2)	226 (78.8)	48 (16.7)
Blinding – participants/personnel	59 (20.6)	132 (46.0)	105 (36.6)
Blinding – outcome assessment	24 (8.4)	111 (38.7)	152 (53.0)
Incomplete data	37 (12.9)	103 (35.9)	141 (49.1)
Selective outcome reporting	40 (13.9)	53 (18.5)	194 (67.6)
Other sources of bias	32 (11.2)	93 (32.4)	162 (56.5)
Overall risk of bias	134 (46.7)	144 (50.2)	9 (3.1)
Funding	8 (2.8)	154 (53.7)	125 (43.6)
Baseline imbalance	13 (4.5)	88 (30.7)	186 (64.8)
Blocked randomization	20 (7.0)	16 (5.6)	22 (7.7)
Early stopping for benefit	2 (0.7)	1 (0.4)	2 (0.7)

Table 3 summarizes the results of the meta-epidemiological analyses based on the combined data from all meta-analyses, including dichotomous and continuous outcomes. No significant differences were observed between trials that were high or unclear versus low risk of bias for any of the domains examined. Results were consistent when examined by type of outcome (i.e., dichotomous and continuous). We conducted a sensitivity analysis examining trials grouped as high risk vs. unclear or low and no differences were found (Table S1 in Appendix S1). Post hoc, based on comments from a peer-reviewer, we conducted sensitivity analyses for high vs. low risk of bias and found no differences (Table S2 in Appendix S1). We conducted meta-regression using the three categories of bias as independent variables and again found no significant differences in effect estimates. Table S3 in Appendix S1 shows results for subgroup analyses based on type of intervention (drug vs. non-drug), type of comparison (placebo/standard care vs. another active intervention), and type of outcome (objective vs. subjective). There were no notable differences within the subgroups between studies at high or unclear versus low risk of bias. Specifically for subgrouping by objective and subjective outcomes, no differences were found between high/unclear and low risk of bias studies for any domain: sequence generation (−0.08 [95% CI −0.71, 0.43] vs. −0.07 [−0.32, 0.17]), allocation concealment (0.25 [−0.06, 0.56] vs. −0.16 [−0.39, 0.06]), blinding of participants/personnel (0.10 [−0.05, 0.24] vs. −0.06 [−0.17, 0.06]), blinding of outcome assessment (0.08 [−0.10, 0.25] vs. 0.07 [−0.18, 0.05]), incomplete outcome data (−0.07 [−0.30, 0.17] vs. −0.15 [−0.43, 0.13]), selective outcome reporting (−0.04 [−0.21, 0.12] vs. −0.08 [−0.20, 0.05]), other sources of bias (0.11 [−0.10, 0.32] vs. −0.08 [−0.32, 0.16]), baseline imbalance (−0.02 [−0.39, 0.35] vs. −0.07 [−0.31, 0.17]), and funding (0.02 [−0.20, 0.24] vs. 0.04 [−0.20, 0.29]).

**Table 3:** Results of meta-epidemiological analysis of bias items and treatment effect estimates.

Domain	Difference of standardizedmean differences	95% CI
Sequence generation	−0.07	−0.22, 0.08
Allocation concealment	0.09	−0.15, 0.33
Blinding (participants/personnel)	0.00	−0.09, 0.09
Blinding (outcome assessment)	−0.00	−0.11, 0.11
Incomplete outcome data	−0.09	−0.26, 0.07
Selective outcome reporting	−0.06	−0.15, 0.04
Other sources of bias	0.05	−0.09, 0.20
Baseline imbalance	−0.07	−0.28, 0.14
Early stopping for benefit	−0.17	−0.49, 0.14
Blocked randomization in unblinded trials	−0.18	−0.47, 0.11
Funding	0.02	−0.13, 0.18

## Discussion

There is a growing body of empirical evidence that aims to quantify the association between different methodological characteristics of randomized trials and treatment effect estimates; however, differences have been found among studies. Some of this variation has been attributed to differences across clinical areas, while another explanation for lack of significant findings or inconsistent findings has been insufficient sample sizes to adequately detect differences. This is the first study to our knowledge that has attempted to quantify bias in a sample of pediatric-only trials. We found no differences in effect estimates based on any of the methodological characteristics that we examined.

The characteristics we examined form the basis for tools that are well-accepted for assessing methodological quality or risk of bias of randomized trials in SRs. However, there is variation in how SR authors handle risk of bias assessments; some may choose to exclude studies outright from a review or meta-analysis based on risk of bias. [29] Given that we did not find any significant differences in effect estimates, we would recommend that trials not be excluded from SRs and/or meta-analyses based on high or unclear risk of bias assessments. Rather, risk of bias should be explored as a potential source of heterogeneity where there is substantial variation observed in effect estimates across studies. Further, the body of evidence being reviewed should be discussed in light of potential methodological weaknesses; however, dismissing large bodies of evidence (for example, all trials without blinding) will severely limit our ability to make recommendations for pediatric care.

Our recommendation not to exclude studies from meta-analyses based on risk of bias is consistent with conclusions from a recently published study reporting on a combined analysis of meta-epidemiological studies. [26], [30] The study examined the influence of sequence generation, allocation concealment, and double-blinding on effect estimates and between-trial heterogeneity based on 1,973 trials included in 234 meta-analyses. The authors found that the methodological characteristics examined were associated with exaggerated treatment effects and increased between-trial heterogeneity. Specifically, the study found exaggerated effect estimates in trials with inadequate or unclear sequence generation (ratio of odds ratio 0.89, 95% credible interval 0.82 to 0.96), allocation concealment (ROR 0.93, 95% CrI 0.87 to 0.99), and double-blinding (ROR 0.87, 95% CrI 0.79 to 0.96). However, when examined by type of outcome (subjective and objective), the results remained significant only for subjective outcomes suggesting an average exaggeration in treatment effect of 17% for sequence generation (CrI 0.74 to 0.94), 15% for allocation concealment (CrI 0.75 to 0.95), and 22% for double-blinding (CrI 0.65 to 0.92). The results were not statistically significant for mortality or other objective outcomes. The authors proposed down-weighting trials at high risk of bias in meta-analyses rather than excluding them completely which results in loss of precision.

Excluding trials completely from an analysis due to risk of bias could leave very little evidence for decision-making. Consistent with previous research, we found that a high proportion of our sample of trials was at high or unclear risk of bias for many domains. [18], [19] Further, only 3% were considered low risk of bias overall which is similar to other reported samples of pediatric trials. [18] Other samples of adult only trials have also found the majority of studies to be at high or unclear risk of bias overall. [31] From an epidemiological perspective, there may be no difference in how typical biases (e.g., selection, performance, detection, attrition, reporting) operate in trials based on population characteristics. Moreover, a recent standard, developed by the international organization StaR Child Health, for minimizing risk of bias in pediatric trials could be equally applied to any trial. [32] Consistent with Savovic et al’s findings, other features may be more salient in assessing possible bias such as choice of outcomes (subjective vs. objective).

There are several limitations to note with the present study. The RCTs included in this study were parallel, superiority design. This may limit the generalizability to other types of trials; however, superiority trials are most common in the scientific literature. The median year of publication in this sample was 1995. Results may differ with more recent trials; however, this does not invalidate the present findings. Newer studies may add to the number of trials assessed as low risk of bias and increase statistical power for the analyses. We based the sample size for the present study on other similar studies; however, this may have limited our ability to identify statistically significant differences. Moreover, Savovic et al. found significant associations primarily in the context of subjective outcomes which represented only a portion of our sample. [26], [30] One of the driving factors for imprecision in the present study was the small number of studies in the low risk of bias (or reference) category. This problem is accentuated for blinding in studies with subjective outcomes.

In summary, we found no significant differences in effect estimates of pediatric randomized trials based on key methodological characteristics. Based on these findings, we recommend that trials not be excluded from SRs and/or meta-analyses based on risk of bias. Rather potential for bias due to methodological characteristics should be considered when exploring heterogeneity and interpreting results.

## Supporting Information

Appendix S1 **Includes Tables S1–S3.** -Table S1. Results of meta-epidemiological analysis of bias items and treatment effect estimates based on sensitivity analyses comparing low/unclear versus high risk of bias. -Table S2. Results of meta-epidemiological analysis of bias items and treatment effect estimates based on sensitivity analyses comparing low versus high risk of bias. -Table S3. Results of meta-meta-analysis of bias items and treatment effect estimates, by sub-groups.
(DOCX)
