# Supplementary material for: Quantifying Bias in Randomized Controlled Trials in Child Health: A Meta-Epidemiological Study
Source: PLoS One. 2014 Feb 4;9(2):e88008. doi: 10.1371/journal.pone.0088008 (PMC3913714; doi:10.1371/journal.pone.0088008)
Supplement: Appendix S1 — Includes Tables S1–S3. -Table S1. Results of meta-epidemiological analysis of bias items and treatment effect estimates based on sensitivity analyses comparing low/unclear versus high risk of bias. -Table S2. Results of meta-epidemiological analysis of bias items and treatment effect estimates based on sensitivity analyses comparing low versus high risk of bias. -Table S3. Results of meta-meta-analysis of bias items and treatment effect estimates, by sub-groups. (DOCX) [file pone.0088008.s001.docx]

**Appendix S1**

**Table S1. Results of meta-epidemiological analysis of bias items and treatment effect estimates based on sensitivity analyses comparing low/unclear versus high risk of bias**

| **Domain** | **Difference of standardized mean differences** | **95% CI** |
| --- | --- | --- |
| Sequence generation | -0.01 | -0.16, 0.15 |
| Allocation concealment | -0.03 | -0.49, 0.13 |
| Blinding (participants/personnel) | 0.23 | -0.10, 0.56 |
| Blinding (outcome assessment) | 0.12 | -0.25, 0.50 |
| Incomplete outcome data | -0.04 | -0.14, 0.05 |
| Selective outcome reporting | -0.09 | -0.19, 0.02 |
| Other sources of bias | -0.06 | -0.29, 0.17 |
| Baseline imbalance | -0.08 | -0.36, 0.20 |
| Blocked randomization in unblinded trials | 0.03 | -0.40, 0.47 |
| Funding | -0.15 | -0.40, 0.10 |

**Table S2. Results of meta-epidemiological analysis of bias items and treatment effect estimates based on sensitivity analyses comparing low versus high risk of bias**

| **Domain** | **Difference of standardized mean differences** | **95% CI** |
| --- | --- | --- |
| Sequence generation | 0.00 | -0.16, 0.17 |
| Allocation concealment | -0.03 | -0.39, 0.33 |
| Blinding (participants/personnel) | 0.45 | -0.03, 0.90 |
| Blinding (outcome assessment) | 0.23 | -0.16, 0.90 |
| Incomplete outcome data | -0.07 | -0.24, 0.10 |
| Selective outcome reporting | -0.11 | -0.23, 0.02 |
| Other sources of bias | -0.07 | -0.32, 0.17 |
| Baseline imbalance | -0.14 | -0.42, 0.14 |
| Blocked randomization in unblinded trials | 0.01 | -0.46, 0.49 |
| Funding | -0.07 | -0.26, 0.12 |

**Table S3. Results of meta-meta-analysis of bias items and treatment effect estimates, by sub-groups**

| **Domain and sub-group** | **Difference of standardized mean differences** | **95% CI** |
| --- | --- | --- |
| Sequence generation |  |  |
| Drug intervention | 0.07 | -0.14, 0.29 |
| Non-drug intervention | -0.16 | -0.38, 0.05 |
| Placebo/no intervention comparison | 0.03 | -0.09, 0.15 |
| Active/mixed comparison | **-0.37** | **-0.82, -0.05** |
| Objective outcome | -0.08 | -0.71, 0.43 |
| Subjective outcome | -0.07 | -0.32, 0.17 |
| Allocation concealment |  |  |
| Drug intervention | 0.03 | -0.31, 0.37 |
| Non-drug intervention | 0.17 | -0.20, 0.53 |
| Placebo/no intervention comparison | 0.02 | -0.19, 0.23 |
| Active/mixed comparison | 1.23 | -1.23, 3.69 |
| Objective outcome | 0.25 | -0.06, 0.56 |
| Subjective outcome | -0.16 | -0.39, 0.06 |
| Blinding (participants/personnel) |  |  |
| Drug intervention | 0.08 | -0.12, 0.29 |
| Non-drug intervention | -0.02 | -0.12, 0.08 |
| Placebo/no intervention comparison | -0.01 | -0.10, 0.09 |
| Active/mixed comparison | 0.04 | -0.20, 0.28 |
| Objective outcome | 0.10 | -0.05, 0.24 |
| Subjective outcome | -0.06 | -0.17, 0.06 |
| Blinding (outcome assessment) |  |  |
| Drug intervention | 0.15 | -0.10, 0.41 |
| Non-drug intervention | -0.06 | -0.14, 0.03 |
| Placebo/no intervention comparison | -0.03 | -0.12, 0.06 |
| Active/mixed comparison | 0.15 | -0.34, 0.65 |
| Objective outcome | 0.08 | -0.10, 0.25 |
| Subjective outcome | 0.07 | -0.18, 0.05 |
| Incomplete outcome data |  |  |
| Drug intervention | 0.02 | -0.20, 0.23 |
| Non-drug intervention | -0.17 | -0.42, 0.07* |
| Placebo/no intervention comparison | -0.02 | -0.13, 0.10 |
| Active/mixed comparison | -0.78 | -2.39, 0.83 |
| Objective outcome | -0.07 | -0.30, 0.17 |
| Subjective outcome | -0.15 | -0.43, 0.13 |
| Selective outcome reporting |  |  |
| Drug intervention | -0.13 | -0.35, 0.10 |
| Non-drug intervention | -0.04 | -0.15, 0.07 |
| Placebo/no intervention comparison | -0.07 | -0.17, 0.03 |
| Active/mixed comparison | 0.10 | -0.20, 0.39 |
| Objective outcome | -0.04 | -0.21, 0.12 |
| Subjective outcome | -0.08 | -0.20, 0.05 |
| Other sources of bias |  |  |
| Drug intervention | 0.17 | -0.21, 0.56 |
| Non-drug intervention | -0.04 | -0.14, 0.05 |
| Placebo/no intervention comparison | 0.02 | -0.27, 0.30 |
| Active/mixed comparison | 0.03 | -0.16, 0.21 |
| Objective outcome | 0.11 | -0.10, 0.32 |
| Subjective outcome | -0.08 | -0.32, 0.16 |
| Baseline imbalance |  |  |
| Drug intervention | 0.23 | -0.37, 0.83 |
| Non-drug intervention | -0.12 | -0.28, 0.03 |
| Placebo/no intervention comparison | -0.02 | -0.27, 0.23 |
| Active/mixed comparison | -0.27 | -0.82, 0.28 |
| Objective outcome | -0.02 | -0.39, 0.35 |
| Subjective outcome | -0.07 | -0.31, 0.17 |
| Funding |  |  |
| Drug intervention | 0.05 | -0.34, 0.44 |
| Non-drug intervention | 0.03 | -0.10, 0.16 |
| Placebo/no intervention comparison | 0.05 | -0.14, 0.23 |
| Active/mixed comparison | -0.04 | -0.23, 0.16 |
| Objective outcome | 0.02 | -0.20, 0.24 |
| Subjective outcome | 0.04 | -0.20, 0.29 |

Bold text indicates statistically significant.

* -0.25 (-0.45, -0.05) for educational asthma/CBT/alarm interventions/secondary prevention for violence
